# Sensorineural hearing loss and risk of stroke: a systematic review and meta-analysis

**DOI:** 10.1038/s41598-021-89695-2

**Published:** 2021-05-26

**Authors:** Masoud Khosravipour, Fatemeh Rajati

**Affiliations:** 1 Student Research Committee, Kermanshah University of Medical Sciences, Kermanshah, Iran; 2 Research Center for Environmental Determinants of Health, Health Institute, Kermanshah University of Medical Sciences, Kermanshah, Iran

**Keywords:** Cardiology, Diseases, Health care, Health occupations, Medical research, Neurology, Risk factors

## Abstract

The aim of this systematic review and meta-analysis study was to clarify the effects of sensorineural hearing loss (SNHL) on the incidence of stroke. In line with this, PubMed, Scopus, Web of Science, and ScienceDirect databases were searched using related keywords and MeSH terms from inception to March 1, 2020. Out of the 1961 initial records, eight cohort studies comprising 4,564,202 participants were included, and their qualities were assessed using the Newcastle-Ottawa Scale (NOS). Then, the random-effects model was used to pool HR (95% CI) for risk of stroke; and heterogeneity was presented with I^2^ index. Subgroup analysis and publication bias tests were performed, and the pooled HR (95% CI) of stroke in SNHL was estimated as 1.31 (1.08, 1.53) for the unadjusted model and 1.33 (1.18, 1.49) for the adjusted model. Subgroup analysis indicates a significantly higher risk of stroke in patients with sudden SNHL (SSNHL) in comparison to age-related HL (ARHL) both in the unadjusted model, [HR = 1.46; 95% CI (1.08, 1.63)] versus [HR = 1.14; 95% CI (0.64, 1.65)], and in the adjusted model, [HR = 1.44; 95% CI (1.15, 1.74)] versus [HR = 1.29; 95% CI (1.24, 1.34)]. Our study showed that patients with SNHL face a higher risk of stroke than those without SNHL. It is necessary to perform hematologic and neurological examinations to help clinicians detect patients who are potentially at risk for stroke.

## Introduction

Hearing loss is the third leading chronic disease worldwide after arthritis and hypertension^1^. The number of people who are living with hearing loss adds up to 466 million; and it is estimated that the figure will reach nearly 630 million by 2030 and could even rise to over 900 million in 2050^2^. Sensorineural hearing loss (SNHL), also known as ‘nerve-related hearing loss’, is defined as being caused by damage to hair cell in the cochlea, spiral ganglia, cranial 8th nerve (auditory nerve), or the central processing auditory centers of the brain. Congenital hearing loss (CHL) is one of the types of SNHL being present at birth and mainly caused by prematurity, maternal morbidity, and genetic disorders^3^. ARHL is an another type of SNHL which is characterized by an increased hearing threshold by more than 25 dB in pure-tone audiometry^2^. Sudden sensorineural hearing loss (SSNHL), usually unilateral, is defined as a hearing loss of 30 dB or more over three sequential frequencies, that develops within 72 h^4,5^. Hearing loss has negative effects on psychological well-being, social communication, self-esteem, and quality of life^6–8^.

Similarly, stroke was the third main cause of mortality in 2017. The total number of deaths from stroke increased from 5.29 million to 6.17 million during 2007–2011^9^.

Stroke is a leading cause of disability and mortality, accounting for seven million deaths in 2012 across the world^10^. The global lifetime risk of stroke for people aged 25 years or older is 24.9%^11^. Although in most cases the pathogenesis of SSNHL is unknown, previous studies suggested that SSNHL might be due to viral or bacterial infections, vascular disorders, ruptured inner ear membrane, and autoimmune diseases^4,12–14^. Among the possible causes of the SSHL, hypothesis of vascular disorders have attracted significant attention in recent years^15^. Accordingly, some recent cohort studies have reported a higher risk of stroke among people who are suffering from SSHL in comparison to the general population^16–22^. A report shows that about 50% of patients experience stroke over 2 years after the SHL^23^. However, studies show that SSNHL increases the risk of ischemic stroke in the general population^21,24^.

Evidence shows a vascular origin in SSHL. Hence, the onset of SSHL may be related to a vascular event such as a minor infraction of the cochlea^25^. The inner ear is especially sensitive to ischemia. Blood flow in the labyrinthine artery is regulated by adrenergic receptors, normal plasma viscosity, and normal platelet function. Cohort studies found a high incidence of cardiovascular risk factors in people with SSHL, possibly in part through a mechanism of microvascular disease leading to stroke^26^.

Previous systematic reviews and meta-analyses have shown the association between hearing loss and a higher risk of cognitive function, cognitive impairment, dementia^27^, falls^28^, and depression. Although some epidemiological studies have documented that hearing loss increases the risk of stroke, a cohort study with adequate sample size conducted by Ciorba et al. (2015) showed that people with hearing loss had a lower risk for stroke^19^. Moreover, those studies that showed SNHL is a risk factor for stroke have failed to report a significant relationship; or due to controversial results between studies, this relationship is not fully understood^19,20^. Therefore, a systematic review and meta-analysis is needed to summarize the available evidence to clarify this association. To this end, we systematically reviewed those studies examined the incidence of stroke after SSHL to estimate the pooled risk of stroke and to determine the influence of some demographic variables, types of hearing loss, and morbidity on the risk of stroke.

## Results

### Selection of studies

**Figure 1:**
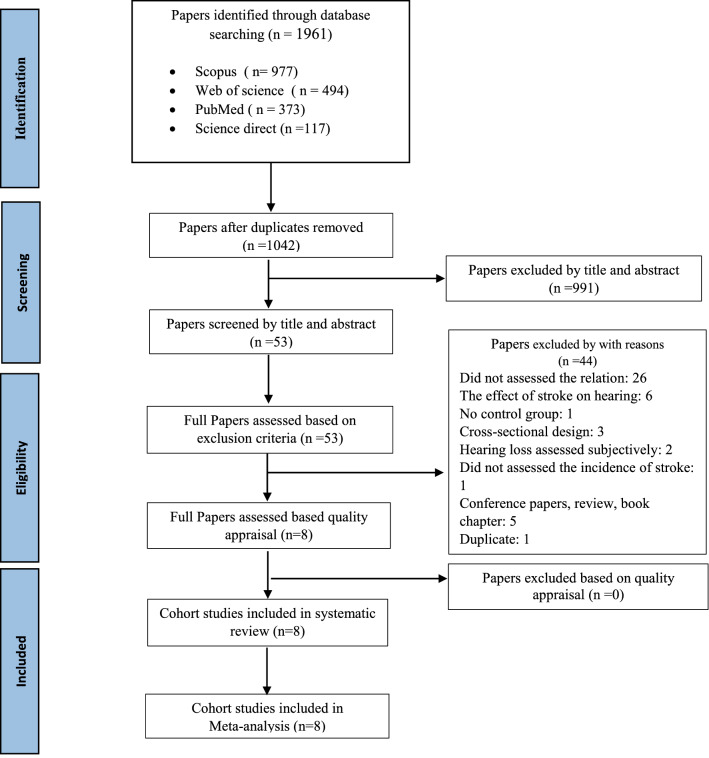
Flow diagram of the systematic review process.

According to the flow diagram illustrated in Fig. 1, after removing the duplicates, we screened 1042 records by title and abstract. Finally, eight cohort studies were included in the study according to the inclusion criteria.

### Study characteristics

As illustrated in Table 1, studies included in our meta-analysis had been conducted in Taiwan (3 articles)^16,17,29^, Korea (2 articles)^21,22^, Australia (1 article), Italy (1 article), and the United States of America (1 article).

**Table 1:** Description of the studies included in the meta-analysis.

Study ID	Country	Study population	Hearing loss assessment				Stroke assessment				Risk measurement			Quality rate
			No	Type	Source (s)	Definition	No	Type	Source (s)	Definition	Index	Crude	Adjusted	
Deal et al. (2018)	USA	Administrative claims data from commercially insured and Medicare Advantage members in a geographically diverse US health plan Follow up: 2000–2016 Gender: M&F Sample size: after 2 (n = 154,414), 5 (n = 44,852), and 10 (n = 4728) years follow up Age: 50 years or older	After 2 years: 77,207 After 5 years: 22,426 After 10 years: 2364	ARHL	Medical record: the presence of at least 2 claims separated by no more than 730 days	ICD-9 codes: V41.2, 306.7, 388.01, 389, 389.1x [excluding 389.12, 389.14], 389.2x)	After 2 years: 3916 After 5 years: 903 After 10 years: 65	Both	Identification algorithms provided by the Centers for Medicare and Medicaid Services Chronic Conditions Data Warehouse	ICD-9 and ICD-10: codes are not reported	RR (95% CI)	NR	1–17	Good
Gopinath et al. (2009)	Australia	Population-based survey among participants of the Blue Mountains Eye Follow up: 1999–2004 Gender: M&F Sample size: 1394 Age: 49 years or older	474	ARHL	Pure-tone Audiometry	The average of frequencies 0.5, 1.0, 2.0, and 4.0 kHz > 25 dB in the better ear	43	Both	MONICA criteria and supporting evidence from computed tomography (CT) and/or magnetic resonance imaging (MRI)	NR	OR (95% CI)	NR	1–2	Good
Kim et al. (2018)	Korea	The Korean National Health Insurance Service-National Sample Cohort (NHIS-NSC) Follow up: 2002–2013 Gender M&F Sample size: 24,720 Age: 5 years or older	4944	SSNHL	Participants who underwent an audiometry examination and were treated with a steroid	ICD-10: H91.2	1038	IS and HS	Medical records	ICD-10: (60-I62 and 163)	HR (95% CI)	Yes	1–2, 14, and 18–23	Good
Kim et al. (2018)	Korea	The Korean National Health Insurance Service (KNHIS) Follow up: 2002–2013 Gender: M&F Sample size: 770 Age: between 45 and 64 years	154	SSNHL	Medical records Pure-tone Audiometry steroid medication	KCD codes: H9120, H9121, H9129, or H810	NR	Both	Medical records	The Korean Classification of Diseases (KCD) codes: I60–I63	HR (95% CI)	Yes	1–2, 18–21, and 24	Good
Chang et al. (2018)	Taiwan	The National Health Insurance Research Database (NHIRD) of Taiwan Follow up: 2002–2009 Gender: M&F Sample size: 216,936 Mean age: 49.5 years	956	SSNHL with vertigo	Medical records	ICD-9: 780.4 for vertigo And 388.2 for SSNHL	8416	Both	Medical records	ICD-9 (430–438)	HR (95% CI)	Yes	1–2, 19–22, and 25–27	Good
Chou et al. (2018)	Taiwan	Dialysis patients Follow up: 1997–2008 Gender: M&F Sample size: 2016 Age: 28% > 64 years	288	SSNHL	Medical records	ICD-9: 388.2	144	IS and HS	Medical records	ICD-9 (430–438)	HR (95% CI)	NR	1–2, 20–22, and 28–32	Fair
Ciorba et al. (2015)	Italy	Population-based survey Follow up: 2001–2012 Gender: M&F Sample size: 4,234,044 participants in the Emilia Romagna region Mean age: 53.3 years for Emilia Romagna region	8188	SSNHL	Medical records	ICD-9: 388.2	112,989	Both	Medical records	ICD-9 codes: 43301, 43311, 43321, 43331, 43381, 43391, 43401, 43411, 43491, and 436	NR	NR	–	Fair
Lin et al. (2008)	Taiwan	NHIRD of Taiwan Follow up: 1998–2003 Gender: M&F Sample size: 7115 Age: 61% ≥ 45 years	1423	SSNHL	Medical records	ICD-9: 388.2	621	Both	Medical records	ICD-9 codes: 430–438	HR (95% CI)	NR	1–2, 18, 20–22, and 24–25	Good

*M* male, *F* female, *SSNHL* sudden sensorineural hearing loss, *ARHL* age-related hearing loss, *IS* ischemic stroke, *HS* hemorrhagic stroke, *ICD* the International Statistical Classification of Diseases and Related Health Problems, *OR* odds ratio, *RR* risk relative, *HR* hazard ratio, *CI* confidence interval, *NR* not reported, *1* age, *2* sex, *3* race/ethnicity, *4* race/ethnicity, *5* net worth, *6* Charison comorbidity index, *7* artery coronary diseases, *8* medical costs, *9* number of inpatient stay, *10* inpatient length, *11* number emergency visits, *12* office visits, *13* dementia, *14* depression, *15* fracture, *16* falls, *17* acute myocardial infarction, *18* income, *19* region of residence, *20* hypertension, *21* diabetes, *22* hyperlipidemia, *23* ischemic heart disease, *24* renal failure, *25* urban status, *26* cardiovascular disease, *27* migraine, *28* duration after the first time hemodialysis, *29* gout, *30* CCT score, *31* antihypertensive drugs, *32* antidiabetic drugs.

The majority of the studies (7 out of 9) had been published in 2018 and 2019. In all cohort studies, at least 2 years' follow-up had been performed; and overall, 5,014,271 participants had been assessed. We found that two studies investigated ischemic and/or hemorrhagic stroke.

In the remaining studies, both ischemic and hemorrhagic stroke were considered as an outcome. In all studies, hearing loss was defined as either ICD-9 code 388.2 or pure-tone audiometry ≥ 25 dB or based on the physician’s diagnosis. For detecting stroke, most studies used ICD-10 (I60–I63) or ICD-9 (430–438) codes.

### Risk of bias within studies

Out of all of the studies included in this meta-analysis, six studies showed an acceptable quality (NOS score = 8) in terms of risk of bias according to NOS (Supplemental Table 1).

### Results of individual studies

**Figure 2:**
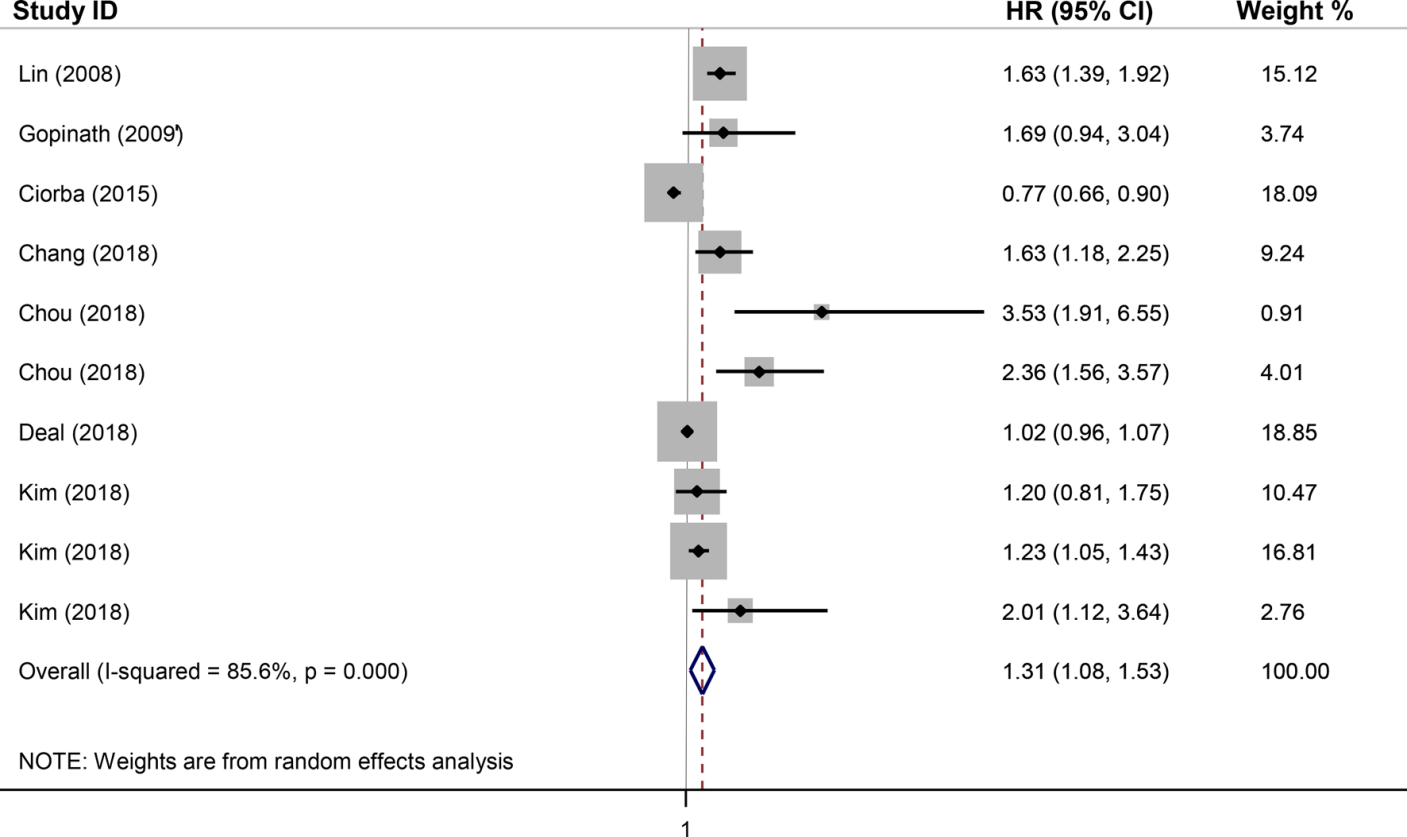
Forest plot for the association between sensorineural hearing loss (SNHL) and the incidence of stroke in the unadjusted model. *HR* adjusted hazard ratio, *CI* confidence interval.

**Figure 3:**
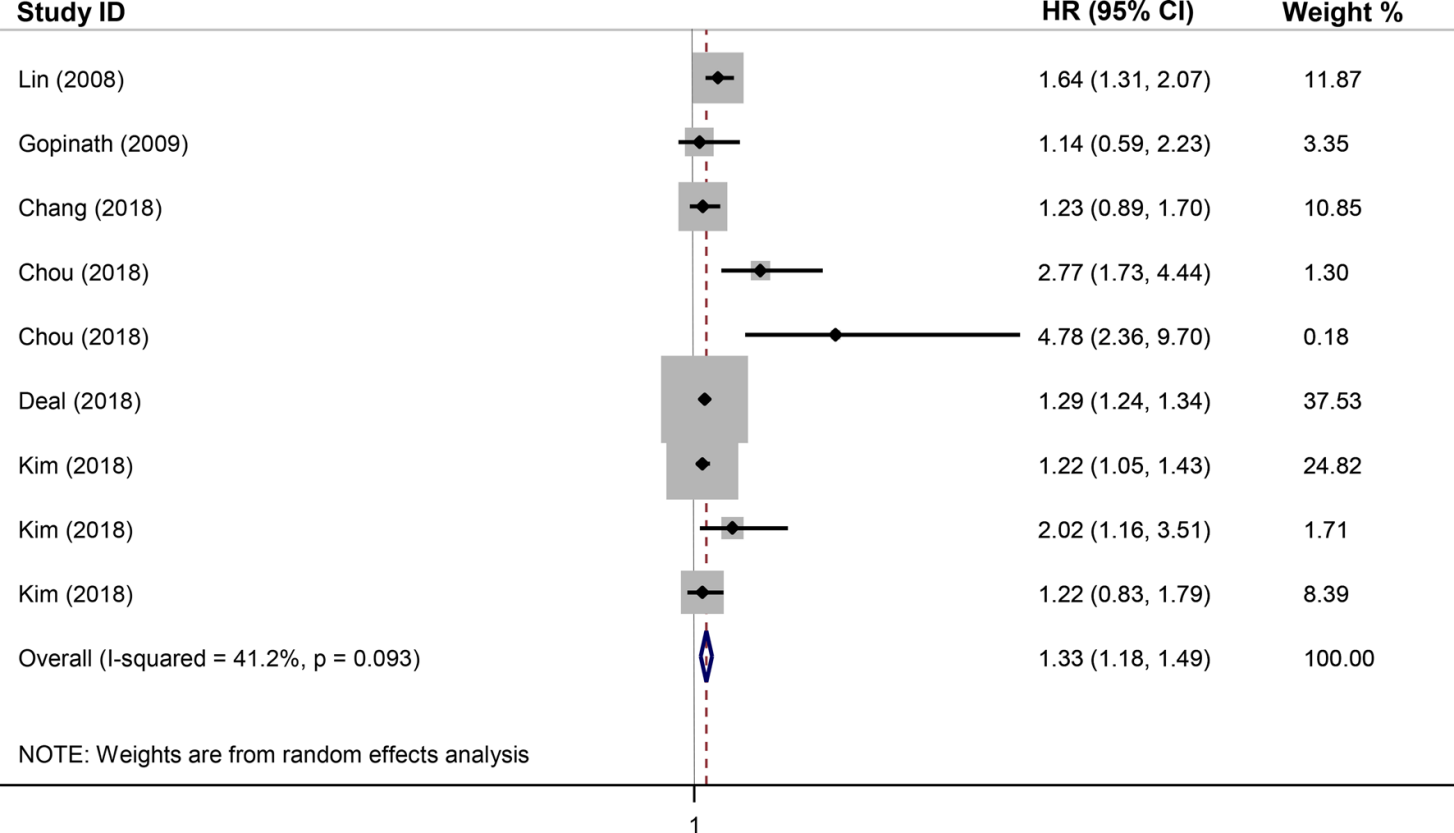
Forest plot for the association between sensorineural hearing loss (SNHL) and the incidence of stroke in the adjusted model. *HR* adjusted hazard ratio, *CI* confidence interval.

We observed that SSNHL had been examined in six studies and ARHL had been assessed in three studies. We found high heterogeneity in the results of studies in the unadjusted model as provided in Fig. 2. In the adjusted model, most of the studies reported a significantly higher risk of stroke in SNHL compared to those with normal hearing status (Fig. 3).

### Synthesis of results

As shown in Fig. 2, the pooled unadjusted HR (95% CI) in subjects with SNHL, in comparison to those with normal hearing status, was estimated as 1.31 (1.08, 1.53) with a high heterogeneity of I^2^ = 85.6% (*p* < 0.001). For the adjusted model, as illustrated in Fig. 3, the pooled HR (95% CI) of stroke was 1.33 (1.18, 1.49) with a moderate and insignificant heterogeneity of I^2^ = 41.2% (*p* = 0.093).

### Risk of bias across studies

**Figure 4:**
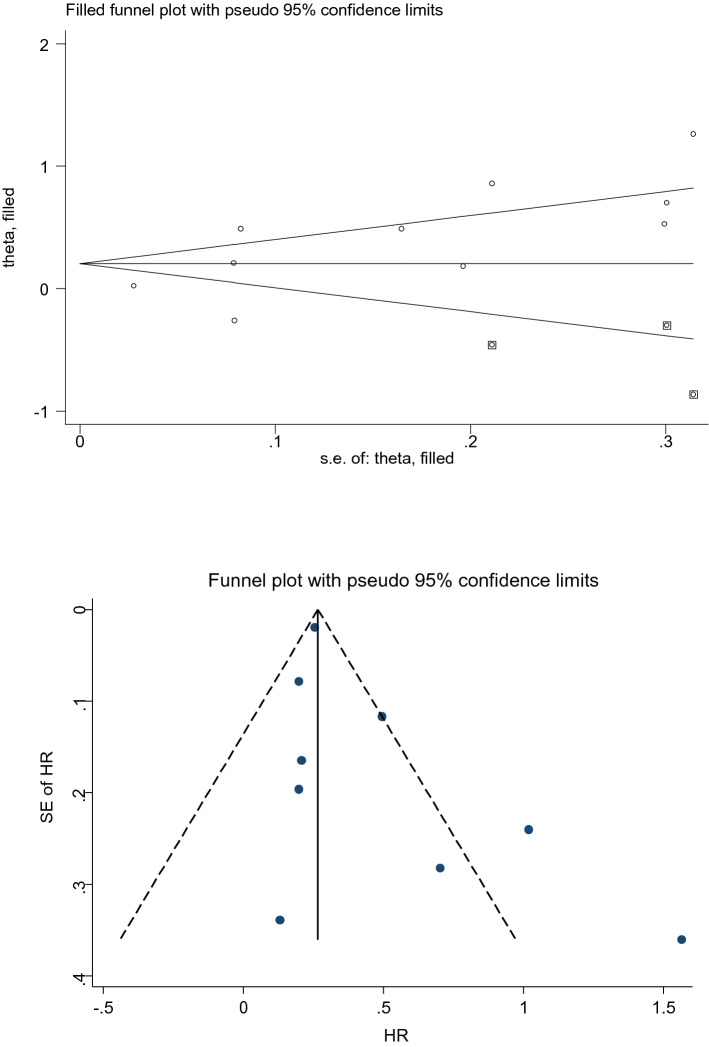
Funnel plots for the association between sensorineural hearing loss (SNHL) and the incidence of stroke in the unadjusted (above) and the adjusted (below) models. *HR* adjusted hazard ratio, *CI* confidence interval.

For meta-analysis on the unadjusted model, we showed a significant publication bias according to Egger's test (*p* = 0.039). We used Fill and Trim method to compute the adopted HR (95% CI). Accordingly, the pooled HR (95% CI) was calculated 1.23 (1.00, 1.50). In contrast, we did not find a significant publication bias according to both Begg’s test (*p* = 0.061) and Egger's test (*p* = 0.120) for the adjusted model. The funnel plots have been provided in Fig. 4 for both the unadjusted and the adjusted models.

### Additional analysis

#### Subgroup analysis

We conducted subgroup analysis according to the types of SNHL (ARHL and SSNHL). As illustrated in Supplemental Figure 1, the pooled unadjusted HR (95% CI) of stroke for the unadjusted model in SSNHL, versus subjects with normal hearing status, was computed as 1.46 (1.08, 1.83). In addition, I^2^ was estimated as 88.5% (*p* < 0.001). For the adjusted model, as provided in Supplemental Figure 2, the pooled HR and I^2^ were calculated as 1.44 (95% CI 1.15, 1.74) and 55.0% (*p* = 0.038), respectively. For ARHL, the pooled estimate of HRs was 1.14 (95% CI 0.64, 1.65), with a heterogeneity of I^2^ = 35.9% (*p* = 0.212) (Supplemental Figure 1). In the adjusted model, the pooled unadjusted HR of stroke was obtained as 1.29 (95% CI 1.24, 1.34), with heterogeneity of I^2^ = 0.0% (0.720) (Supplemental Figure 2).

#### Sensitivity analysis

Sensitivity analysis was examined according to demographic variables and potential risk factors. Because only those studies that addressed SSNHL had reported HR (95% CI) for the above-mentioned variables, we recruited these studies in the sensitivity analysis. As shown in Supplemental Table 2, sensitivity analysis according to the types of participants indicated that SSNHL subjects with comorbidities, including kidney disease and vertigo, had a significantly higher risk of stroke in the adjusted model. In addition, there was no statistically significant difference between men and women in the incidence of stroke. However, being man increases the risk of stroke up to % 47 (HR 1.14; 95% CI 0.80, 1.47). People aged 45–64 and older were strongly more likely to be at risk of stroke compared to people aged less than 45 years (HR 5.87; 95% CI 3.56–8.18). While people with hypertension, hyperlipidemia, gout, or renal diseases had no more likelihood of risk of stroke, the probability of stroke incidence was higher among individuals who had more than one morbidity (HR 1.97; 95% CI 1.25, 5.69).

## Discussion

To the best of our knowledge, this study was the first systematic review and meta-analysis study to quantify and clarify the possible association between SNHL and the incidence of stroke. We found a significantly higher risk of stroke in subjects with SNHL in comparison to those with normal hearing status in both the unadjusted and the adjusted models. Similarly, subgroup analysis based on the type of SNHL showed a significantly higher risk of stroke in subjects suffering from SSNHL in both the unadjusted and the adjusted models. We also observed a similar result in subjects with ARHL in the adjusted model. Although biological pathways undertaking the association between SSNHL and the risk of stroke remained unknown and not fully understood, several pathophysiological mechanisms have been proposed, ranging from vascular disorders and membrane rupture to viral infection and autoimmunity^14,30–33^. However, vascular involvement in the inner ear in cases of SSNHL acquired more attention^18,34,35^. It is supposed that cochlear hair cells have high metabolic activity and are only supplied by the terminating spiral modiolar artery, a branch of the anterior inferior cerebellar artery, which is strikingly vulnerable to ischemia^36,37^. Literature shows that the cochlear function starts to be impaired after one minute of anoxia^38^, and it is not reversible after one hour of vessel obstruction^32,39^. Vascular occlusion may impair the cochlear perfusion within terminal capillary beds in the same way in coronary, cerebral, and peripheral regions. While a review study shows that the ischemic stroke syndrome leads to hearing loss, other prospective studies, such as those included in the current study, approve that SNHL may be a risk factor for the incidence of stroke. These findings strengthen the hypothesis of the vascular origin of SNHL. Hence, the association between SNHL and stroke is not a direct causality; rather it is likely that such an association functions as a confounding variable due to vascular impairment. Therefore, it can be claimed that SNHL is a marker for stroke incidence or previous stroke rather than being a risk factor for it.

Therefore, whereas SSNHL can be considered as a sign of stroke, it is observed that SSNHL is seriously ignored in stroke preventive care. In one of the included studies, conducted by Lin et al. (2008), it was observed that half of the subjects with SSNHL were admitted to hospital due to stroke about 2 years after SSNHL^12^. Besides, in another study, conducted by Ciorba et al. (2015), the mean interval between SSNHL and stroke was 54.2 to 41.2 months^19^. Some studies have also investigated the association between SSNHL and cardiovascular risk factors^22^. The results of these studies indicated that there is a higher risk of SSNHL in subjects with diabetes^40^, metabolic syndrome^41^, and renal failure^42^. This may confirm the vascular involvement pathways in the incidence of SSNHL. Another hypothesis could be the involvement of autoantibodies in hearing loss^43^, as the association of different autoantibodies with stroke has recently been recognized^44^.

In addition, we pointed out interesting results in the sensitivity analysis. According to the types of the participants, we found that subjects with comorbidities along with SSNHL in both the unadjusted and the adjusted meta-analysis had a significantly higher risk of SSNHL stroke. Evidence has been documented regarding the significant effects of comorbidities on the incidence of stroke^45^. However, the risk of stroke was significant for individuals who suffered from SSNHL without any morbidities in the adjusted model.

We also showed that there was no sex difference in stroke incidence after hearing loss. However, in some of the included studies, men had a higher risk of stroke compared to women. Furthermore, we found that aging plays a dramatically significant role in the association between SSNHL and stroke. In that way, subjects aged 45–64 and older, compared to subjects aged less than 45 years, had a significantly higher risk of stroke, approximately 6 and 15 times, respectively. This finding emphasizes the role of age as an important confounder. In fact, it is well-established that aging can be associated with structural and functional alterations in the cardiovascular system such as increased arterial stiffness, hypertrophy, impaired endothelial function, and altered left ventricular (LV) diastolic function, whereby increasing the risk of developing cardiovascular diseases and stroke^46–49^. It is necessary that more studies be performed among middle and lower-middle age population so that the association between SSNHL and stroke will be clarified better. One of the strengths of our study was that the effects of hypertension, diabetes, renal disorders, and hyperlipidemia were taken into account as confounders. It is well-known that comorbidities are important risk factors for stroke^50^. Given that it is reported in previous studies that subjects with diabetes^51^, metabolic syndrome^52^ and chronic renal failure^42^ had a higher risk of SNHL, these confounders can play a significant role in the association between SSNHL and stroke. We observed that patients with ARHL like SSNHL patients, faced a significantly stronger risk of stroke than subjects with normal hearing loss. However, this association highly depends on the small number of studies included in the analysis as well as the age of the participants. In all of the studies included in our meta-analysis, the minimum age of the subjects was 49 years. Although the stroke is less likely to occur in younger age groups, more studies need to be performed on younger subjects to obtain a comprehensive understanding of the association between SNHL and stroke.

Although most of the included studies had a cohort design with good quality, sample size, and follow up duration (≥ 2 years), some limitations should be noted. First, for SSNHL, we ran two independent meta-analyses for studies reporting adjusted and unadjusted effect sizes. Although this condition can be associated with increasing our analysis validity, we found a significant publication bias for our analysis on the adjusted model. Second, we observed in the two studies that the subjects had other diseases in addition to SSNHL. These studies significantly reported a higher risk for both the unadjusted and the adjusted models. However, the sensitivity analysis according to the types of participants indicated a significantly higher risk of stroke in SSNHL subjects in the adjusted model. Third, most of the studies did not consider the effects of additional confounders on the association between hearing loss and stroke. As a result, in this study, the role of smoking^53,54 ^, obesity^55^, physical activity^56^, occupational and environmental exposures such as shift work and noise, air pollution, all being associated with both stroke and hearing loss, has not been addressed. Fourth, most of the included cohort studies had a retrospective method.

We reviewed all available studies to assess the relationship between SNHL, including both SSNHL and ARHL, and the incidence of stroke. We found that subjects with SNHL have a higher risk of stroke. In addition, subgroup analysis according to the types of SNHL indicated that there is a higher risk of stroke in individuals with SSNHL compared to subjects with ARHL. This meta-analysis suggests that SSNHL may be considered as an early warning of stroke. However, more studies are needed to obtain a comprehensive result considering potential confounders such as smoking habits, body mass index (BMI), diet plan, and occupational and environmental exposures. Diagnostic assessments should be considered the early diagnosis of stroke in patients who have lost their hearing.

## Methods

### Protocol

We conducted the preferred reporting items for systematic reviews and meta-analyses (PRISMA) statement in the study^57^ with the explanation and elaboration of the PRISMA statement^57^.

### Eligibility criteria

In the current study, the PICOTS (population, intervention, comparison, outcome, publication time, and study design) was defined as follows. The study population was considered as any subjects who had been investigated in the included studies. We did not apply any specific age, gender, and ethnicity criteria. Exposure group was defined as the subjects who had SNHL, whether ARHL or SSNHL. We did not consider any intervention either, because the aim of our study was to estimate the effects of the SNHL on the incidence of stroke. The comparison group was defined as the subjects with a normal hearing status and without any stroke history. Furthermore, the incidence of stroke after SNHL was defined as the outcome. We included only those studies that had a cohort design; and no limitation of publication date was considered.

### Information sources

To investigate the association between SNHL and the incidence of stroke, we selected the following databases for the identification of relevant publications: PubMed (https://www.ncbi.nlm.nih.gov/pubmed/), Scopus (https://www.scopus.com/), Web of Science (https://www.webofknowledge.com/), and ScienceDirect (https://www.sciencedirect.com/). Furthermore, we screened the references of the selected studies to find any relevant studies that may not have been detected by searching.

### Search

We searched the mentioned databases from inception to March 1, 2020, using keywords and terms such as “hearing loss”, “hearing impairment”, and “stroke”. We download Endnote files of attained records for further assessment.

### Study selection

After importing the records to Endnote version X8.1 and removing the duplicate records, both authors of the present article cross-checked the documents by title and abstract. First of all, the studies that had not investigated the association between hearing loss and the incidence of stroke were excluded. Such an exclusion was also applied to the studies that had assessed the impacts of stroke on the incidence of hearing loss. Moreover, the studies that had estimated the risk of stroke through the 10-year atherosclerotic cardiovascular disease (ASCVD) tool and had not considered the incidence of stroke were also removed^58^. Then, we retrieved the full text of the selected studies, and the data are extracted from eligible reports. Cross-sectional studies were also discarded; and the studies that had subjectively assessed hearing loss (self-reported) were eliminated. We also excluded studies that did not allocate a comparison group who had normal hearing status. However, we included research letters or editorials which reported the results of primary research.

### Data collection process

We assessed all of the included studies carefully. Each study was reviewed by both of the authors and then the main characteristics of the studies were recorded using Microsoft Excel version 2013.

### Data items

The study ID (name and publication year), the country where the study had been conducted, the characteristics of the study population (age, sex, and sample size), the types of both SNHL (ARHL and SSNHL) and stroke, and the estimated risk of stroke after SNHL were all collected for each study (Table 1).

### Risk of bias in individual studies

We assessed the selected studies in terms of risk of bias. We used the Newcastle-Ottawa Scale (NOS) developed for observational studies. The NOS is a user-friendly scale which is largely used due to recommendations from the Cochrane Collaboration^59^. The scale ranged between 0 (the lowest quality) and 9 (the highest quality) points. Each author independently reviewed the included studies and scored each study and solved any discrimination.

### Summary measures

All of the included studies had cohort design and objectively investigated the impacts of SNHL on the incidence of stroke. Five out of six cohort studies having investigated SSNHL reported adjusted hazard ratios (HRs) and 95% confidence intervals (CIs) for estimating the risk of stroke in subjects with hearing loss versus normal hearing groups. However, in another study, only the number of subjects with and without SSHNL and the incidence of stroke in each group had been reported. Therefore, we had to calculate unadjusted relative risk (RR) and 95% CI for it. We considered OR and HR the same, because the incidence of stroke was less than 10% and the rate of HR/RR was less than three^45^. Similarly, for ARHL, one study reported the adjusted HR (95% CI) and another study adjusted OR (95% CI). In the present study, we ran two independent meta-analyses for the unadjusted and adjusted models. It should be noted that for ARHL, one study reported HR (95% CI) of stroke after 2, 5, and 10 years’ follow-up. Hence, it is supposed that the subjects having been followed for 10 and 5 years were also in the 2 years’ follow-up analysis. Therefore, first the three HRs of 10, 5, and 2 years’ follow-up were combined using fixed model meta-analysis and then the obtained pooled HR was included in our meta-analysis.

### Synthesis of results

The random-effects model through a generic inverse-variance method was used to calculate the pooled HRs for the incidence of stroke in both the unadjusted and the adjusted models^60^. Heterogeneity was presented with calculated I^2^ index, and values of 0%, 25%, 50%, and 75% represent no, low, moderate, and high heterogeneity, respectively^61^. The *p* < 0.05 was considered to test the null hypothesis in all analyses. The analysis of data was conducted in Stata version 11.

### Risk of bias across studies

We applied Egger's and Begg’s tests to investigate publication bias^62,63^ with a *p*-value of < 0.05. In addition, visual inspection of the funnel plot was performed.

### Additional analyses

Subgroup analyses based on the types of SNHL, including ARHL and SSNHL, were performed (Supplemental Figure 1, Figure 2). Moreover, sensitivity analysis based on age, gender, and comorbidity was conducted only for SSNHL due to the availability of these data in the studies (see Supplemental Table 2).

## Supplementary Information

Supplementary Information.

## Data Availability

Data is available upon request.
