# Supplementary material for: Sensorineural hearing loss and risk of stroke: a systematic review and meta-analysis
Source: Sci Rep. 2021 May 26;11:11021. doi: 10.1038/s41598-021-89695-2 (PMC8155183; doi:10.1038/s41598-021-89695-2)
Supplement: Supplementary file 1 — Supplementary Information. [file 41598_2021_89695_MOESM1_ESM.docx]

**Sensorineural hearing loss and risk of stroke: A systematic review and meta-analysis**

***Masoud Khosravipour^1^, Fatemeh Rajati^2🞾^***

Students Research Committee, Kermanshah University of Medical Sciences, Kermanshah, Iran.

***Email:*** [masoudkhosravipour74@gmail.com](mailto:masoudkhosravipour74@gmail.com)

***^2^*Fatemeh Rajati:***

Research Center for Environmental Determinants of Health, Health Institute, Kermanshah University of Medical Sciences, Kermanshah, Iran.

***Email:*** [f.rajati@kums.ac.ir](mailto:f.rajati@kums.ac.ir), *****Corresponding Author**

***Phone:*** +988338350177

**Postal address:** Kermanshah University of Medical Sciences, Kermanshah, Iran.

**Running head:** Sensorineural hearing loss and risk of stroke

**Sources of financial support:** Kermanshah University of Medical Sciences

**Supplemental table 1**. The Newcastle-Ottawa Assessment Scale for studies

| **Quality assessment** | Chang 2018 | Chou  2018 | Kim  2018 | Lin  2008 | Ciorba  2015 | Deal  2018 | Gopinath  2009 | Fang 2018 | Kim  2017 |
| --- | --- | --- | --- | --- | --- | --- | --- | --- | --- |
| Exposed cohort representative? | ■ | □ | ■ | ■ | ■ | ■ | ■ | ■ | ■ |
| Selection of non-exposed cohort? | ■ | □ | ■ | ■ | ■ | ■ | ■ | ■ | ■ |
| Ascertainment of exposure? | ■ | ■ | ■ | ■ | ■ | ■ | ■ | ■ | ■ |
| Outcome at baseline? | ■ | ■ | ■ | ■ | ■ | □ | ■ | □ | ■ |
| Controls for important factors? | ■ | ■ | ■ | ■ | □ | ■ | ■ | ■ | ■ |
| Controls for other confounders? | ■ | ■ | ■ | ■ | □ | ■ | ■ | ■ | □ |
| Assessments of outcome? | ■ | ■ | ■ | ■ | ■ | ■ | ■ | ■ | ■ |
| Adequacy of follow-up duration? | ■ | ■ | ■ | ■ | ■ | ■ | ■ | □ | ■ |
| Adequacy of lost to follow up? | ■ | □ | □ | ■ | ■ | ■ | □ | □ | ■ |
| **Quality score** | 9 | 6 | 8 | 9 | 7 | 8 | 8 | 6 | 8 |
|  | Good | fair | good | good | fair | good | good | fair | good |

**Supplemental Figure 1**. Forest plot for the association between sensorineural hearing loss (SNHL) and the incidence of stroke according to types of SNHL in the unadjusted model. HR, adjusted hazard ratio; CI, confidence interval.

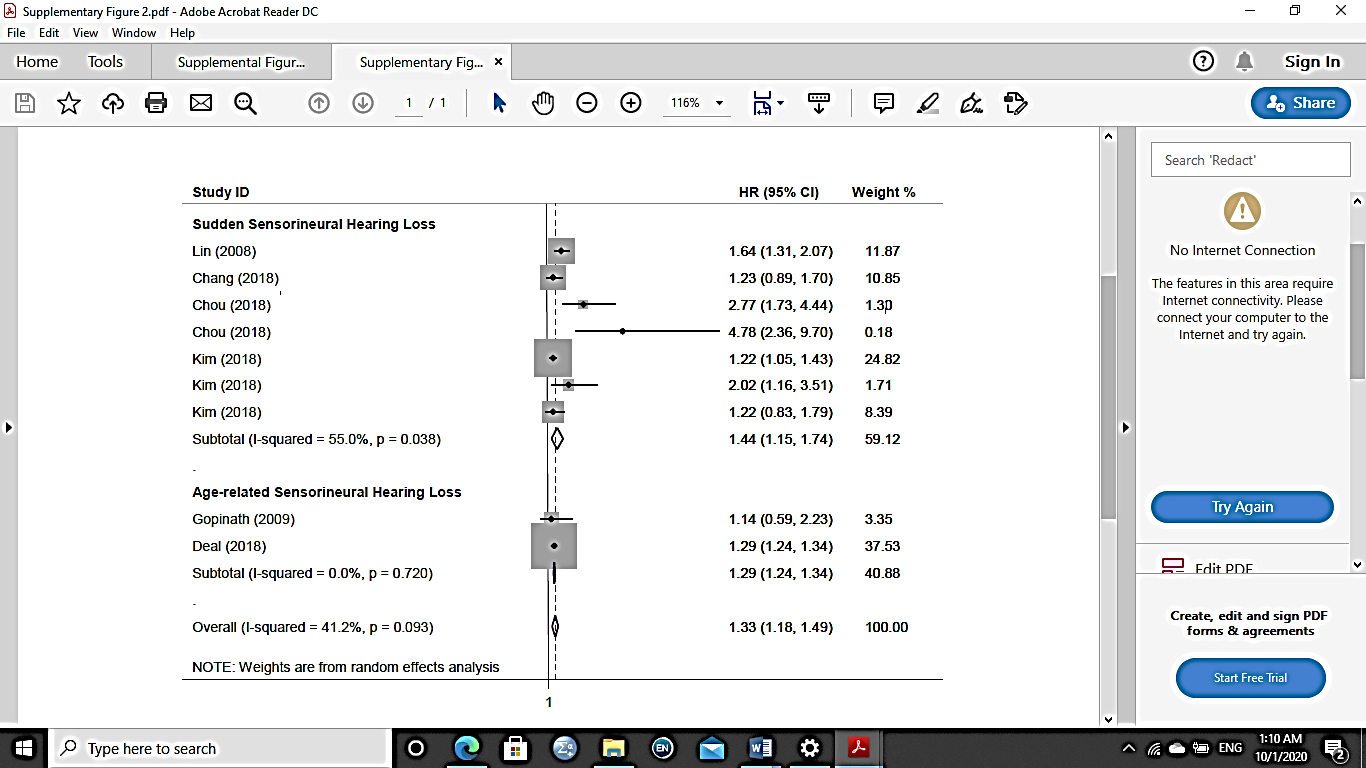
 **Supplemental Figure 2**. Forest plot for the association between sensorineural hearing loss (SNHL) and the incidence of stroke according to types of SNHL in the adjusted model. HR, adjusted hazard ratio; CI, confidence interval.

**Supplemental Table 2.** Sensitive analysis for SSNHL

| Variables | | | Types of  stroke | Number of  Studies (estimations) | HR(95%CI) | Heterogeneity | |
| --- | --- | --- | --- | --- | --- | --- | --- |
|  |  |  |  |  |  | I^2^ | p-value |
| Types of participants | Only SSNHL | Adjusted | B,H,I | 3(4) | 1.38(1.10,1.65) | 43.4% | 0.151 |
|  |  | Unadjusted | B,H,I | 4(6) | 1.16(0.81,1.51) | 88.8% | <0.001 |
|  | Patients with SSNHL | Adjusted | B,H,I | 2(4) | 1.91(1.04,2.77) | 64.4% | 0.038 |
|  |  | Unadjusted | B,H,I | 2(4) | 1.96(1.45,2.46) | 19.6% | 0.292 |
| Gender | | male vs, female | B,H,I | 2(3) | 1.14(0.80,1.47) | 43.0% | 0.173 |
| Age | | < 45 vs. 45-64 | B | 2(2) | 5.87(3.56,8.18) | 0.0% | 0.888 |
|  |  | < 45 vs. 64 < | B | 2(3) | 15.35(10.46,20.24) | 0.0% | 0.606 |
| Diabetes | | Yes vs. No | B,H,I | 3(3) | 1.62(0.44,2.28) | 81.7% | 0.004 |
| Hypertension | | Yes vs. No | B,H,I | 3(3) | 3.03(0.29,5.77) | 93.1 | < 0.001 |
| Hyperlipidemia | | Yes vs. No | B,H,I | 3(3) | 1.37(0.54,2.21) | 0.0% | 0.461 |
| Gout | | Yes vs. No | H,I | 1(2) | 1.42(0.20,2.65) | 0.0 | 0.570 |
| Renal | | Yes vs. No | B | 1(1) | 0.9(0.42,1.94) | NA. | NA. |
| Comorbidity | | Yes vs. No | B | 1(1) | 3.7(2.15,6.35) | NA. | NA. |
| Combined | | Yes vs. No | B,H,I | 3(13) | 1.97(1.25,5.69) | 77.7% | < 0.001 |

**Abbreviation**: HR, hazard ratio; CI, confidence interval; I, ischemic strike; H, hemorrhagic stroke; B, both types; SSNHL, sudden sensorineural hearing loss.
